# Combining QTL-seq and linkage mapping to fine map a wild soybean allele characteristic of greater plant height

**DOI:** 10.1186/s12864-018-4582-4

**Published:** 2018-03-27

**Authors:** Xiaoli Zhang, Wubin Wang, Na Guo, Youyi Zhang, Yuanpeng Bu, Jinming Zhao, Han Xing

**Affiliations:** Soybean Research Institute, National Center for Soybean Improvement, Key Laboratory of Biology and Genetic Improvement of Soybean (General, Ministry of Agriculture), State Key Laboratory of Crop Genetics and Germplasm Enhancement, Jiangsu Collaborative Innovation Center for Modern Crop Production Nanjing Agricultural University, Nanjing 210095, China, Nanjing Agricultural University, Nanjing, 210095 China

**Keywords:** Plant height, Wild soybean, QTL-seq, Linkage mapping, Candidate gene

## Abstract

**Background:**

Plant height (PH) is an important agronomic trait and is closely related to yield in soybean [*Glycine max* (L.) Merr.]. Previous studies have identified many QTLs for PH. Due to the complex genetic background of PH in soybean, there are few reports on its fine mapping.

**Results:**

In this study, we used a mapping population derived from a cross between a chromosome segment substitution line CSSL3228 (donor N24852 (*G. Soja*), a receptor NN1138–2 (*G. max*)) and NN1138–2 to fine map a wild soybean allele of greater PH by QTL-seq and linkage mapping. We identified a QTL for PH in a 1.73 Mb region on soybean chromosome 13 through QTL-seq, which was confirmed by SSR marker-based classical QTL mapping in the mapping population. The linkage analysis showed that the QTLs of PH were located between the SSR markers BARCSOYSSR_13_1417 and BARCSOYSSR_13_1421 on chromosome 13, and the physical distance was 69.3 kb. RT-PCR and sequence analysis of possible candidate genes showed that *Glyma.13 g249400* revealed significantly higher expression in higher PH genotypes, and the gene existed 6 differences in the amino acids encoding between the two parents.

**Conclusions:**

Data presented here provide support for *Glyma.13 g249400* as a possible candidate genes for higher PH in wild soybean line N24852.

**Electronic supplementary material:**

The online version of this article (10.1186/s12864-018-4582-4) contains supplementary material, which is available to authorized users.

## Background

Soybean (*Glycine max* (L.) Merr.) is an important economic crop and provides most of the plant protein and oil used for human consumption. It is vital to increase world-wide soybean production and seed/pod yield potential [1]. The ideal plant type is the genetic basis of high yield potential. PH is one of the most important traits of plant type and is positively related to yield under the lodging resistance condition in soybean [2]. Wild soybean (*Glycine soja* Sieb. et Zucc.) with the characteristic of greater PH is a valuable genetic resource for improving cultivated soybean [3–5]. However, most of the yield-related traits in soybean, such as PH and days to flowering, are complex quantitative traits governed by multiple major or minor genes/QTLs (quantitative trait loci) and affected by the environment. Thus, identification and fine mapping/map-based cloning of the genes underlying these important agronomic traits have been the most effective approaches for molecular breeding [1, 6].

Over the past few decades, rapid progress in DNA markers and their linkage maps has facilitated the identification, localization, and dissection of loci conferring PH and its related trait DTF(Days to flowering), and numerous QTLs for both traits have been identified in soybean with primary mapping populations (F_2_, F_2:3_, RILs, etc.) [7, 8]. According to the SoyBase database (http://www.soybase.org/), a total of 255 PH QTLs have been detected in soybean, distributed among 20 linkage groups [9]. Based on the information on these QTLs, the mapping accuracy is not high, and all of them were mapped in large genomic regions due to the genetic background noise [10, 11] and limited recombination in the primary mapping population. Meanwhile, the method of QTL mapping is also highly time-consuming and laborious. QTL mapping is difficult to accurately identify minor QTL and epistasis QTL. Hence, it will affect subsequent QTL fine mapping, cloning and molecular breeding. Therefore, it is necessary to develop a new secondary mapping population and to fully detect polymorphic markers between parents in the target region, which is of great significance for QTL mapping and related gene cloning.

Chromosome segment substitution lines (CSSLs) are ideal materials for genome research, particularly for QTL mapping and cloning. Using the secondary mapping population derived from CSSLs and a recurrent parent, many important trait QTLs were fine mapped and map-based cloned in a crop [12–14]. For example, *Dt1* and *Dt2*, regulating stem growth habit, and *GmILPA1*, affecting the leaf petiole angle, have been breakthroughs of fine mapping and map-based cloning in soybean [15, 16]. However, the QTL of PH is still not fine mapped in soybean, which limits its use in marker-assisted breeding. To facilitate the fine mapping of complex quantitative traits, we constructed a CSSL population. An elite cultivar NN1138–2 was used as a recipient parent, and a wild soybean N24852 was used as a donor parent. Backcrossing for 2–5 times, two or more generations of selfing and two applications of marker-assisted selection were constructed [17].

QTL mapping is labor-intensive, time-consuming and sometimes costly [18]. Bulked-segregant analysis (BSA) provides a simple and effective alternative to identify molecular markers linked to target genes or QTLs affecting a trait of interest by genotyping only a pair of bulked DNA samples from two sets of individuals with distinct or opposite extreme phenotypes [19]. With the rapid development of next-generation sequencing (NGS) technologies, new strategies have been proposed to take advantage of the power of BSA and NGS-aided high-throughput genotyping, which have been demonstrated to identify major QTLs in yeast [20, 21], *Arabidopsis thaliana* [22], rice [23, 24], and sunflower [25]. More recently, Takagi et al. described the QTL-seq approach for the rapid mapping of QTLs in rice by whole genome resequencing of DNA bulks of phenotypic extremities [26].

In the present study, to analyze the genetic difference between a higher PH CSSL3228 and its recipient parent NN1138–2 with lower PH, a secondary mapping population was first constructed. Secondly, QTL-seq was carried out by whole genome re-sequencing of DNA bulks of phenotypic extremities, and a genomic region harboring the major PH QTLs was detected, which was confirmed and fine mapped using classical linkage mapping. Thirdly, the expression pattern of the genes in the mapping regions were investigated through quantitative RT-PCR. The results from the study provide preliminary evidence that a 69.3 kb region on chromosome 13 harbored major QTLs of PH and that *Glyma.13 g249400* is a possible candidate gene.

## Results

### The performance of CSSL3228, NN1138–2 and their derived populations

**Fig. 1:**
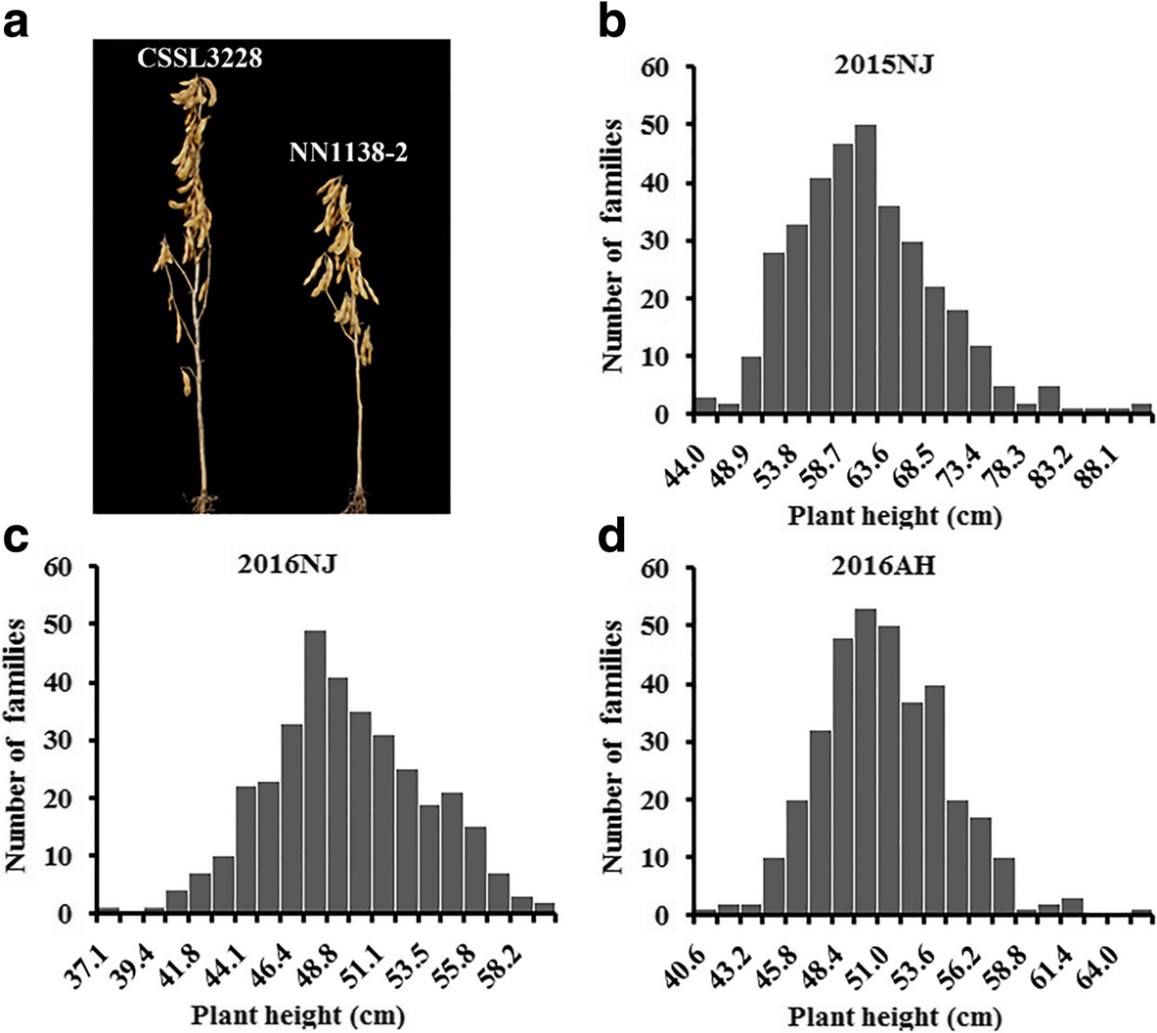
Frequency distribution of plant height (PH) among 349 F_2:3_ and F_2:4_ families along with two parents. **a** represents the chromosome segment substitution line CSSL3228 and its recurrent parent NN1138-2; **b** represents the distribution of plant height (PH) among 349 F_2:3_ families in the environment of 2015NJ; and **c** and **d** represent the distribution of PH among 349 F_2:4_ families in the environments of 2016NJ and 2016AH, respectively

The PH was evaluated for the tested material, F_2:3_ and F_2:4_ families derived from each of the 349 F_2_ plants, in 2015 and 2016, in Nanjing and Anhui, respectively, along with their two parents, CSSL3228 (P1) and NN1138–2 (P2). The frequency distributions of PH in the mapping population are shown in Fig. 1b–d, continuous variations were detected among the mapping populations, with PH ranges of 43.30–92.30 cm, 40.42–66.42 cm and 37.00–60.44 cm in the environments of 2015 Nanjing (2015NJ), 2016 Nanjing (2016NJ), and 2016 Anhui (2016AH), respectively. As shown in Table 1, stable and significant differences were observed in PH between P1 and P2 and among their derived populations of 349 F_2:3_ families and 349 F_2:4_ families across the 3 environments. The heritability of PH was 88.97% in 2016NJ and 85.24% in 2016AH, which suggests that genetic variation accounted for a major part of the phenotypic variance in the populations. A joint ANOVA (Analysis of Variance) of the data from multiple environments showed that the variations among genotypes, environments, and genotype × environment interactions were all significant (Additional file 1: Table S1). Therefore, QTL mapping was performed separately based on individual environments.

**Table 1:** Statistical measures of PH estimated in parental phenotype and 349 families of mapping population (CSSL3228 × NN1138-2) in 3 environments

Env.	Mapping population	Parents		Mean ± S.D.	Range	CV (%)	*h*^*2*^ (%)
		CSSL3228	NN1138-2				
2015NJ	F_2:3_ families	70.50 ± 1.71	53.33 ± 1.11	61.27 ± 7.87	43.30–92.30	-	-
2016NJ	F_2:4_ families	54.00 ± 0.82	44.17 ± 0.90	49.22 ± 3.95	37.00–60.44	8.49	88.97
2016AH	F_2:4_ families	57.00 ± 1.29	46.17 ± 1.07	50.72 ± 3.59	40.42–66.42	8.18	85.24

Env. represents environment, in which 2015NJ, 2016NJ and 2016AH represent the three environments that were used to evaluate the phenotype of the mapping populations; CV represents the coefficient of variation; and *h*^*2*^ represents heritability

#### QTL-seq identified a major PH QTL on chromosome 13

Based on phenotyping data of PH among the F_2:3_ mapping population, two extreme pools were prepared and subjected to the QTL-seq. A total of 20 F_2_ plants with greater PH, ranging from 58.67 cm to 88.33 cm, and 20 F_2_ plants with lesser PH, ranging from 35.00 cm to 64.67 cm, were selected and referred to as HPH and LPH, respectively.

**Fig. 2:**
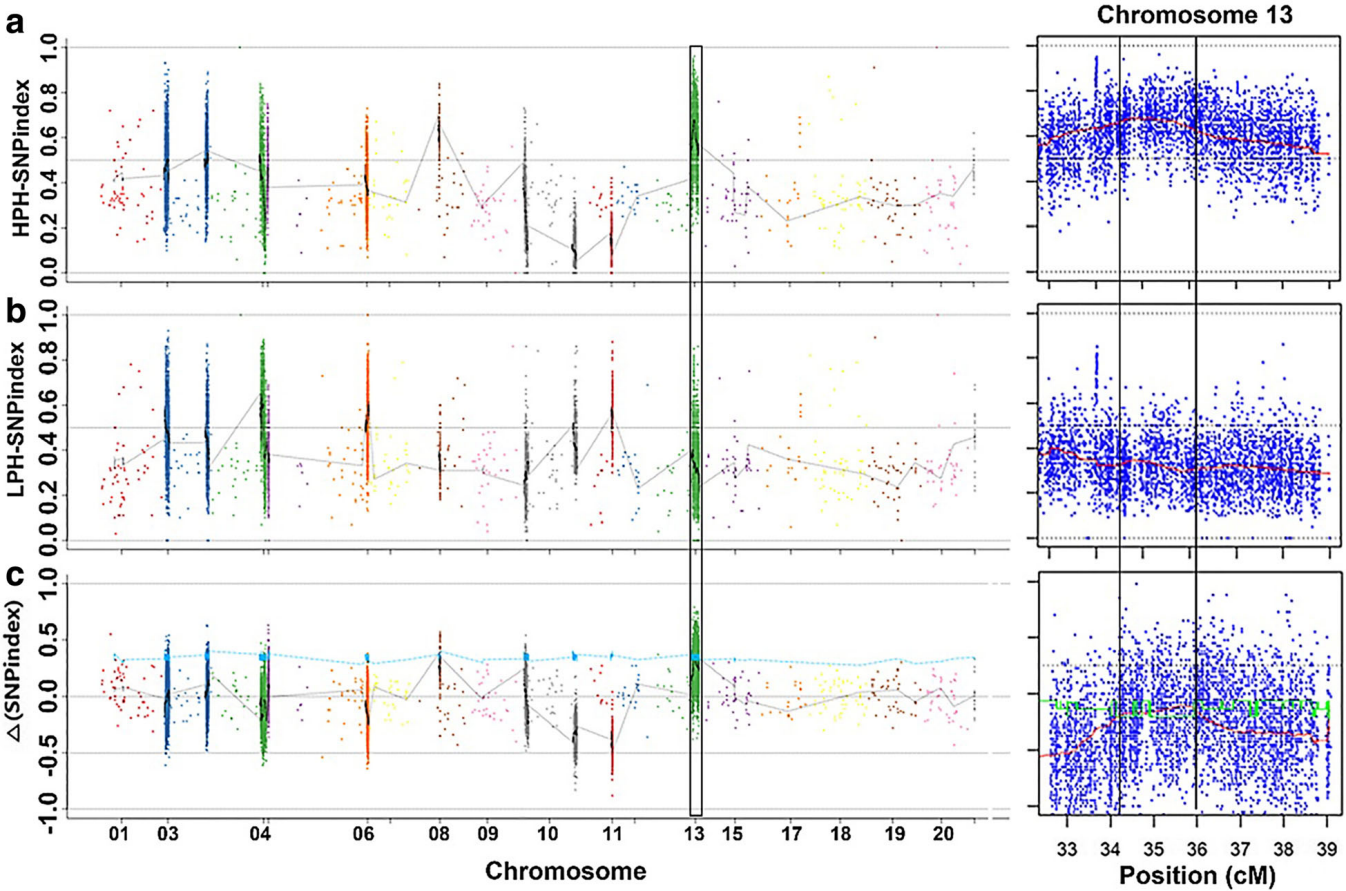
Identification of PH QTL *qPH13.1* at the 34.20–35.93 Mb region on chromosome 13 by QTL-seq. **a**, **b** and **c** represent the single nucleotide polymorphism (SNP)-index plots of HPH-pool and LPH-pool, Δ (SNP-index) plot of 20 soybean chromosomes (left) and 32.5–39.0 Mb region on chromosome 13 (right), respectively, based on QTL-seq analysis. Y-axis represents the SNP-index calculated based on a 1 Mb interval with a 1 kb sliding window. The Δ (SNP-index) graph was plotted with statistical confidence intervals under the null hypothesis of no QTL (*P* < 0.05, blue and red), and one candidate major genomic interval (34.20–35.93 Mb) (marked by one rectangle in the left and bordered by two vertical black lines in the right) harboring a PH QTL (*qPH13.1*) was defined using the criteria of SNP-index near to 1 and 0 in HPH and LPH, respectively

A SNP-index was calculated for each identified SNP. SNP-index values across the genome were computed at a 1 Mb interval using a 1 kb sliding window and were plotted for the HPH-pool (Fig. 2a) and LPH-pool (Fig. 2b). By combining the information on SNP-index in the HPH-pool and LPH-pool, Δ (SNP-index) was calculated and plotted against the genome positions (Fig. 2c). At the 95% statistical level, only one genomic region of 1.73 Mb on chromosome 13 from 34.20 to 35.93 Mb had a Δ (SNP-index) value that was significantly different from 0 (Fig. 2c). These results indicated that there was a major QTL related to PH at the 1.73 Mb region of chromosome 13, named *qPH13.1*.

### Narrowing down *qPH13.1* to a 69.3 kb interval by linkage analysis

**Fig. 3:**
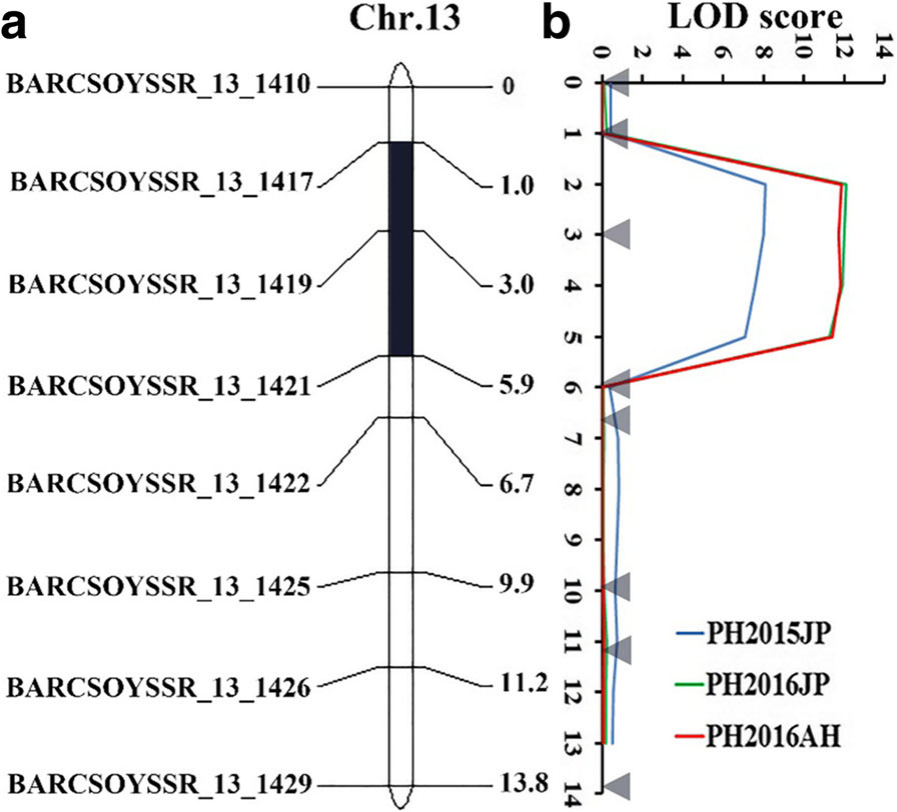
Validation of PH QTLs detected by QTL-seq through linkage mapping. **a** represents the linkage map of the target region detected by QTL-seq constructed by the Join Map v4.0 software, and **b** represents the distribution of the LOD scores for plant height over the target region detected by QTL-seq on chromosome 13. LOD scores were calculated independently by ICIM for the three environments. The LOD score for 2015NJ is in blue, 2016NJ is in green, and 2016AH is in red

To confirm and narrow down the PH QTLs detected by QTL-seq, we conducted classical bi-parental QTL analysis with 349 F_2_ plants and their derived F_2:3_ and F_2:4_ families. Among 112 SSR markers in the target major QTL region detected by QTL-seq, 8 polymorphic SSR markers were between CSSL3228 and NN1138–2 (Table 2). The 8 SSR markers were applied to genotype the 349 F_2_ plants. For a combination of genotypes and their PH values, linkage mapping was carried out using ICIM mapping software. A major QTL for PH was physically located in a region of 69.3 kb between the SSR markers BARCSOYSSR_13_1417 and BARCSOYSSR_13_1421 on chromosome 13 across the 3 environments (Table 3, Fig. 3), and the allele from wild soybean had a positive effect on PH. Days to flowering was also used to complete QTL mapping in the target region in the mapping population, as there was a difference between the two parents. However, no QTL was detected (Additional file 2: Figure S1). These QTL mapping results support a major QTL for PH in the genomic interval of 69.3 kb on chromosome 13.

**Table 2:** The 8 polymorphic markers used to narrow down the PH QTLs detected by QTL-seq

Marker	Chr.	Start^a^	End	Primer sequence
BARCSOYSSR_13_1410	Gm13	35508707	35508744	U: GCGTATTCCCTTAACAAAATTAAAGTTTCAC
				L: GCGCGTCAGCCTAACAAAAAGAATAAAAT
BARCSOYSSR_13_1417	Gm13	35668210	35668255	U: CAAAAGAAACCTCATTCCTGC
				L: AACGTTAATGCGTGGAGATT
BARCSOYSSR_13_1419	Gm13	35698358	35698407	U: ACGAAGGTGGAATGAGGATG
				L: TCTCATTCGTGCCCTGTAGT
BARCSOYSSR_13_1421	Gm13	35737431	35737464	U: TCTCAAGTCATTTTAATTACCGC
				L: AAAATTTTCATTCACTTGGATTTT
BARCSOYSSR_13_1422	Gm13	35738647	35738680	U: TGAATTTAATAGTCATGCACGGA
				L: CCCAAAAATCATAGTTGGTTG
BARCSOYSSR_13_1425	Gm13	35791995	35792026	U: TTTCTCCCCATGTCAAATTGT
				L: TCTCCTCAAATTCCAACTTTTG
BARCSOYSSR_13_1426	Gm13	35797040	35797063	U: CTCCACCCTGCGGTAATAAA
				L: TTTGGGGATAGCCAAAGAAA
BARCSOYSSR_13_1429	Gm13	35848793	35848826	U: CCTCAATGTTTTGAGCGATTC
				L: GGTTCAAATTGGCGGTTTTA

^a^Location in draft genome assembly of Glyma 2.0

**Table 3:** QTL mapping of plant height on chromosome 13 in 3 environments

Env.	Chr.	Interval	Position(bp)	LOD	*R*^2^(%)	Additive effect	Dominant effect
2015NJ	13	SSR_13_1417-SSR_13_1421	35668210-35737464	8.11	10.31	3.26	1.02
2016AH	13	SSR_13_1417-SSR_13_1421	35668210-35737464	11.86	14.67	1.86	0.04
2016NJ	13	SSR_13_1417-SSR_13_1421	35668210-35737464	14.35	14.94	2.03	0.38

*Chr.* chromosome

### Analysis of the candidate genes of *qPH13.1* through RT-qPCR

According to the Williams 82 reference genome sequence [27] (Version Glyma 2.0), a total of 12 genes were contained in the genomic region of *qPH13.1*, which was narrowed down to a 69.3 kb interval by QTL fine mapping (Table 4). To identify the candidate gene for *qPH13.1*, the expression patterns of the 12 annotated genes were tested in three tissues (leaf, stem and shoot apical meristem) of NN1138–2 and CSSL3228, at four growth developmental stages—V1(Fully developed leaves at unifoliolate nodes), V3(Three nodes on the main steam with fully developed leaves beginning with the unifoliolate nodes), R1(One open flower at any node on the main stem) and R2(Open flower at one of the two uppermost nodes on the main stem with a fully developed leaf)—through RT-qPCR [28].

**Table 4:** The function annotation of the 12 genes contained in the interval of *qPH13.1*

Gene ID	Position (bp)	Function annotation
*Glyma.13g248600*	35666896..35668024	Late embryogenesis abundant protein
*Glyma.13g248700*	35668869..35672143	S-locus Lectin Protein Kinase
*Glyma.13g248800*	35677452..35681265	S-locus Lectin Protein Kinase
*Glyma.13g248900*	35685233..35690583	receptor kinase 3
*Glyma.13g249000*	35692094..35696834	S-locus lectin protein kinase
*Glyma.13g249100*	35700102..35701373	Unknown
*Glyma.13g249200*	35701896..35706080	receptor kinase 3
*Glyma.13g249300*	35712020..35716076	S-locus lectin protein kinase
*Glyma.13g249400*	35719291..35721124	plant U-box 8
*Glyma.13g249500*	35721467..35721860	Unknown
*Glyma.13g249600*	35726229..35729214	Quinone reductase family protein
*Glyma.13g249700*	35731494..35733559	Quinone reductase family protein

**Fig. 4:**
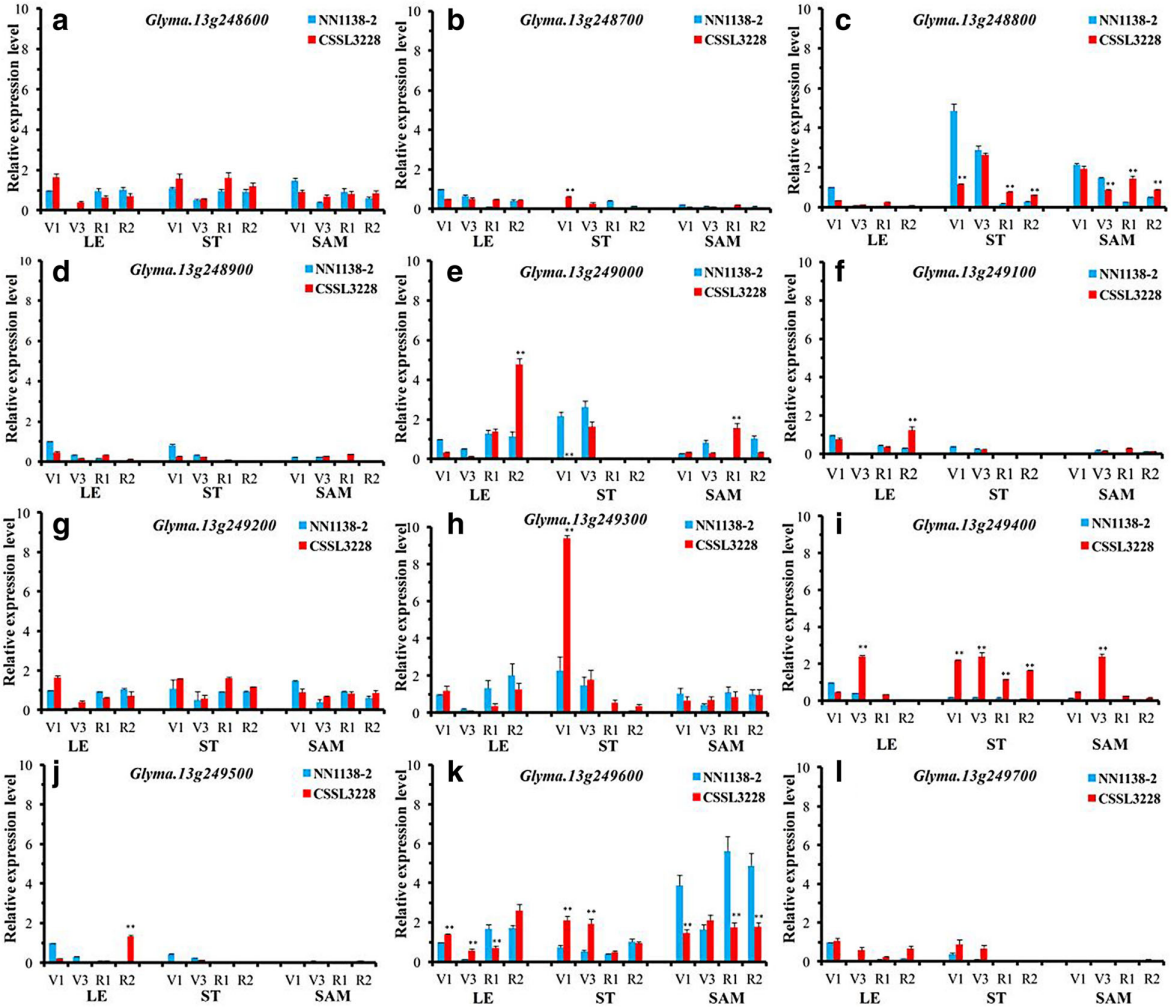
Expression of 12 candidate genes in leaves, stems and shoot apical meristems of NN1138-2 and CSSL3228 at four growth developmental stages. **a**-**l** represent the expression patterns of 12 candidate genes in three tissues of NN1138-2 and CSSL3228, including leaf (LE), stem (ST) and shoot apical meristem (SAM), at four growth developmental stages of V1, V3, R1, and R2, respectively; Asterisks on the top of bars indicate statistically significant differences (*p* < 0.01) between NN1138-2 and CSSL3228 within the pair of comparison of the combination of tissue and growth developmental stage

As shown in Fig. 4 and Additional file 3: Table S2, a total of five annotated genes, *Glyma.13 g248800, Glyma.13 g249000, Glyma.13 g249300, Glyma.13 g249400* and *Glyma.13 g249600*, displayed strongly different expressions of more than two-fold in at least one of the three tissues, including leaf (LE), stem (ST) and shoot apical meristem (SAM), during the four different growth stages. Among them, *Glyma.13 g248800, Glyma.13 g249000, Glyma.13 g249300, Glyma.13 g249400* and *Glyma.13 g249600*, showed different expressions between NN1138–2 and CSSL3228 at a significance level of *P* = 0.01. During all four tested growth stages, there was pronounced differentially expressed in ST and SMA of higher PH parental genotype CSSL3228 and lower PH genotype NN1138–2. Therefore, the differential expression of these genes in two parents (CSSL3228 and NN1138–2) provides a strong support for their possible use as candidate genes for plant height.

### Candidate genes sequence analysis

**Fig. 5:**
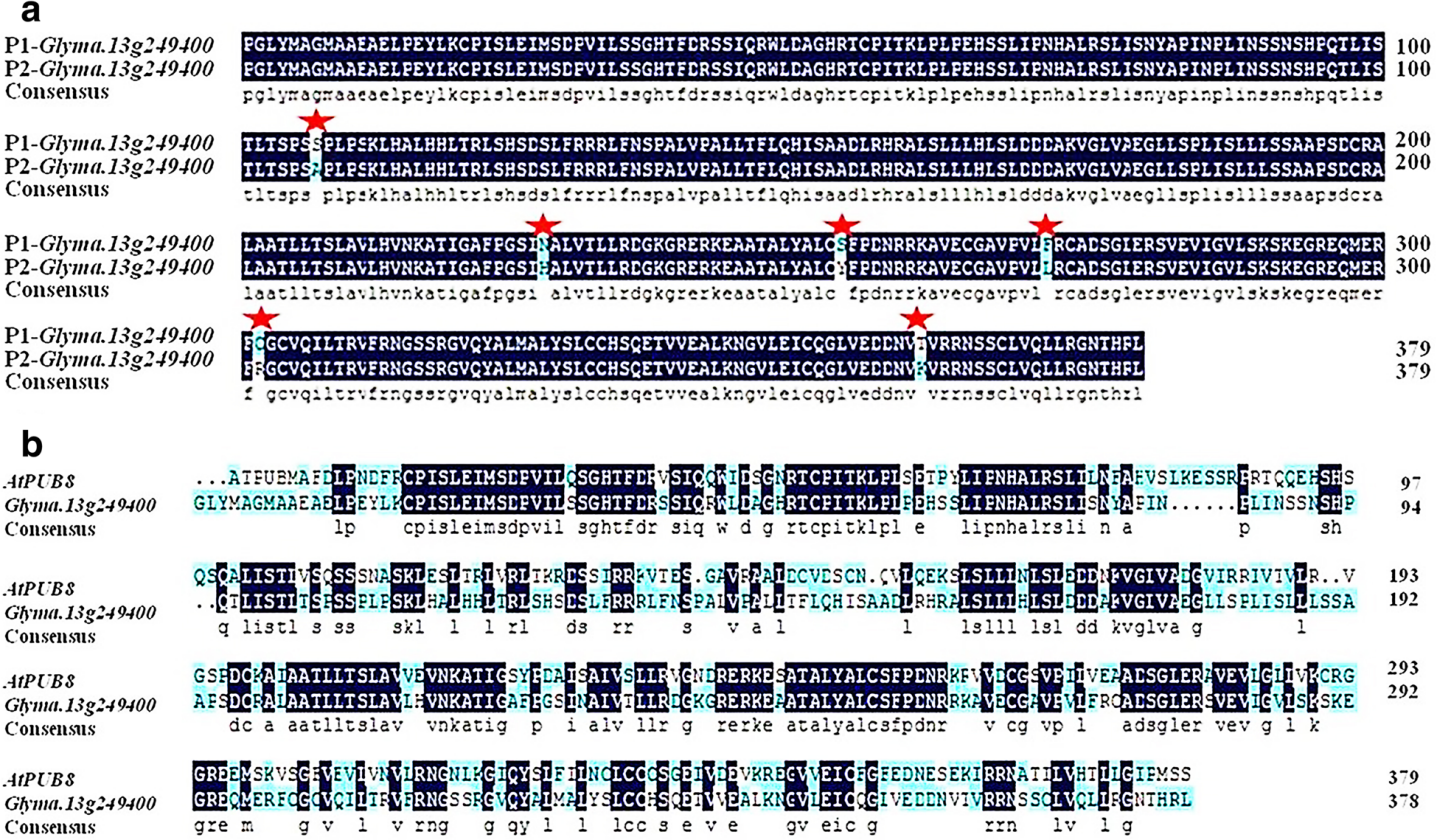
Multiple sequence alignment depicting the amino acid sequence conservation of *Glyma.13g249400*. **a** represent multiple sequence alignment depicting the amino acid sequence conservation of P1(CSSL3228) *Glyma.13g249400* gene with P2(NN1138-2) gene (*Glyma.13g249400*) in soybean, **b** represent multiple sequence alignment depicting the amino acid sequence conservation of soybean Glyma.13g249400 gene with its orthologous gene (*AtPUB8*) in *Arabidopsis thaliana* (At)

In order to further clarify the sequence differences of the five candidate genes (*Glyma.13 g248800, Glyma.13 g249000, Glyma.13 g249300, Glyma.13 g249400* and *Glyma.13 g249600*), we sequenced the genes in P1 (CSSL3228) and P2 (NN1138–2) (Fig. 5a, Additional file 4: Figure S2, Additional file 5: Figure S3 and Additional file 6: Figure S4). Because the sequence of *Glyma.13 g249000* has continuous structure and poly structure, which leads to disorder of sequencing results, and sets of peaks, the study did not find the *Glyma.13 g249000* in the parent sequence. We found that there was only one different sequence between the two parents in the gene *Glyma.13 g249400*, which existed 6 differences in the amino acids encoding between the two parents (Fig. 5a). Therefore, among the four genes obtained, *Glyma.13 g249400* is most likely to be involved in the regulation of plant height in soybean. To analyze the genetic mechanism of plant height in soybean, we still need to further study these two genes.

## Discussion

Soybean was domesticated in China approximately 6000–9000 years ago [29]. Due to bottlenecks and human selection, cultivated soybean has much lower genetic diversity than its wild counterpart [4, 5]. Wild soybean (*Glycine soja* Sieb. et Zucc.), with characteristics of high reproductivity, high number of pods, high protein content, adaptability to various stresses, etc., is acknowledged to be the wild progenitor of the cultivated soybean (*Glycine max* (L.) Merr.) [30]. One of the important goals of genomic and genetic studies in crops is to identify the favorable alleles or genes from unique germplasm resources, such as wild accessions, that can be used to improve agronomic traits and thereby agricultural productivity. In cultivated soybean, some excellent genes in wild soybean have been detected and used for genetic improvement [31]. For example, a total of 142 (times) QTLs for 22 morphological and yield-related traits were reported by using eight inter-specific mapping populations, including A81–356022 × PI468916, NN1138–2 × N24852 (Additional file 7: Table S3), containing the wild positive alleles in protein concentration [32], increased seed yield [33] etc. A total of 64 (times) QTLs were detected for the six seed quality traits (Additional file 8: Table S4), among which approximately 30 *G. soja* alleles had associated positive effects. Although wild soybean is associated with low oil content, *G. soja* alleles, which could improve oil content, have been detected in wild soybean N24852 [34]. A total of 25 QTLs for resistance to diseases and pests and tolerance to stress traits were detected in wild soybean (Additional file 9: Table S5). Among them, 14 *G. soja* alleles had associated positive effects. These results demonstrate the potential of identifying positive alleles in the exotic germplasm of soybean.

PH is an important agronomic trait with yield contribution in soybean [2]. Wild soybean, with a characteristic of greater PH, might contain positive alleles. Previous research has identified many PH QTLs in cultivated soybean by using primary populations, which promotes our comprehensive understanding of the genetic basis. However, it is difficult to fine map and map-based clone a QTL by using a primary population, such as F_2_ and RILs, due to whole genome segregant in these populations [35–37]. Therefore, the development of new sub-populations, such as mapping near-isogenic lines (NILs) and chromosome segment substitution lines (CSSLs), is necessary. In our research, a set of CSSL populations were developed by using wild soybean as a donor parent to mine its excellent genes. In the population, a CSSL with greater PH was found. To explore the wild allele of greater PH, a secondary mapping population was developed using the greater PH CSSL and recurrent parent NN1138–2. Subsequently, a major QTL of PH was detected, conformed and fine mapped into a 69.3 kb region on chromosome 13. Regarding previous research, QTLs were also reported by Gai et al. [37] but were mapped into a large region by an RIL population derived from NN1138–2 and Kefeng No.1. Meanwhile, the major PH QTLs were found to be pleiotropic and associated with plant height, which explained the strong correction of phenotype between them [38].

A combination of QTL and linkage mapping could effectively detect and fine map the QTL of interest. In the present study, major PH QTLs were identified and mapped into a 69.3 kb genomic region on chromosome 13 by using an F_2_ secondary mapping population via whole-genome NGS-based high-throughput QTL-seq. The QTL-seq-detected PH QTLs based on SNP-index were further validated by SSR marker-based traditional QTL mapping (at higher LOD > 14) (Fig. 3) and were mapped between the SSR markers BARCSOYSSR_13_1417 and BARCSOYSSR_13_1421, which suggests the validity and robustness of QTL-seq as a strategy for quick and efficient scanning of major QTLs on a genome-wide scale in soybean. The advantages of QTL-seq vis-a-vis other available traditional QTL mapping approaches [39–41] to identify major QTLs governing seedling vigor, blast resistance and flowering time in crops, including rice and cucumber, have been recently reported [42, 43]. This method took advantage of the high-throughput whole genome resequencing and bulked-segregant analysis (BSA). In addition, the use of a SNP-index allowed accurate quantitative evaluation of the frequencies of the parental alleles as well as the genomic contribution from the two parents to F_2_ individuals. These features of QTL-seq make it a quicker and more efficient method to identify genomic regions harboring major QTLs of the target gene.

In the present paper, the major PH QTLs were delimited into a 69.3 kb physical interval on chromosome 13 (Table 3) with the strategy of combining QTL-seq and classical QTL analysis. There were 12 predicted genes in this region. Among the 12 genes, the function annotation (Table 4) and expression pattern (Additional file 3: Table S3, Fig. 1a) of the *Glyma.13 g249000* and *Glyma.13 g249400* genes suggested that it could be a candidate gene for *qPH13.1*. According to the functional annotation, *Glyma.13 g249000* belongs to S-locus lectin protein kinase family protein gene, which can be involved in signal transduction, anti-retrogradation reaction and pathogen reaction in plants, which is of great significance to plants. There are currently no reports related to plant growth and development [44]. In addition, sequence analysis also found that there were 6 amino acid differences in the gene *Glyma.13 g249400* between the two parents (Fig. 5a). *Glyma.13 g249400* shared 53% amino acid sequence identity with *AtPUB8*, and the expression level of *Glyma.13 g249400* in CSSL3228 plants was higher than that in NN1138–2 plants (Figs. 4i and 5b). There are three go functional annotation results of Glyma.13 g249400, GO:0000151 (ubiquitin ligase complex), GO:0004842 (ubiquitin-protein ligase activity) and GO:0016567 (protein ubiquitination). Collectively, this gene is ubiquitin ligase-related, and is currently not commented on KEGG pathway. U-box ubiquitin ligases belong to a ubiquitin ligase. Plant U-box (*PUB*) proteins are a small family of proteins with the U-box motif [45–48]. The U-box comprises ca. 70 amino acids and resembles a modified RING finger that forms a similar structure stabilized by salt-bridges and hydrogen bonds [46]. *PUBs* have E3 activity [49, 50]. *PUB* E3s are involved in diverse biological processes such as development, self-incompatibility, and response to hormones. They are widely connected with plant stress response [50–53]. Therefore, it is reasonable to postulate that *Glyma.13 g249400* is the candidate gene for PH in soybean. However, further evidence is needed to functionally validate this hypothesis.

## Conclusions

In this study, we identified a QTL for PH in a 1.73 Mb region on soybean chromosome 13 through QTL-seq, which was confirmed by SSR marker-based classical QTL mapping in the mapping population. The linkage analysis showed that the QTLs of PH were located between the SSR markers BARCSOYSSR_13_1417 and BARCSOYSSR_13_1421 on chromosome 13, and the physical distance was 69.3 kb. To identify the candidate gene for *qPH13.1*, the expression patterns of the 12 annotated genes were tested in three tissues (leaf, stem and shoot apical meristem) of NN1138–2 and CSSL3228, at four growth developmental stages—V1, V3, R1 and R2—through RT-qPCR. A total of five annotated genes, *Glyma.13 g248800*, *Glyma.13 g249000*, *Glyma.13 g249300*, *Glyma.13 g249400* and *Glyma.13 g249600*, displayed strongly different expressions of more than two-fold in at least one of the three tissues, including leaf (LE), stem (ST) and shoot apical meristem (SAM), during the four different growth stages. According to candidate genes sequencing analysis, in which *Glyma.13 g249000* was not included, only *Glyma.13 g249400* had sequence differences in the two parents among other four genes, and there are six differences in the amino acids encoding. Therefore, *Glyma.13 g249400* and *Glyma.13 g249000* may play a positive role in the formation of plant height in soybean.

## Methods

### Plant materials and phenotypic evaluation

CSSL3228 is a chromosome segment substitution line developed in a previous study by introgressing a few chromosomal segments of a wild soybean (*Glycine soja* Sieb. et Zucc.) N24852 in maturity group (MG) III into an elite cultivar in MG III. The genetic background of NN1138–2 includes three generations of backcrossing and four generations of selfing [17]. The CSSL3228 showed significantly higher PH than the recurrent parent NN1138–2 based on over 2 years’ phenotypic evaluations in Nanjing, China (N 31.2, E 118.4) (Fig. 1a).

To uncover the genetic basis of PH, an F_2_ population composed of 349 plants was constructed by using CSSL3228 as the male parent and NN1138–2 as the female parent. The F_2_ population and F_2:3_ families derived from each of the 349 F_2_ plants, along with their parents, were grown in June 2014 and 2015 in Nanjing, respectively, in a single plot without replication and in a complete randomized block design with three replications, one row per plot, 20 plants per row, 10 cm between plants within each row and 50 cm between rows. Then, the F_2:4_ families derived from each of the 349 F_2_ plants, along with their parents, were planted at two different geographical locations, Nanjing and Anhui, in June 2016. The planting manner of each plot was the same as that of the F_2_ population and F_2:3_ families previously described. The field management was under normal soybean production conditions.

In the present paper, PH was measured as the length from the ground to the terminal bud of a plant at maturity. For phenotyping the F_2_ population, five plants were selected at random from each of the F_2:3_ and F_2:4_ families, and the mean of the F_2:3_ and F_2:4_ families was representative of the phenotype of the F_2_ plants. The diverse statistical attributes, including the coefficient of variation (CV), broad-sense heritability (*h*^*2*^), frequency distribution, correlation coefficient and analysis of variance (ANOVA) of PH in the mapping population, were analyzed using IBM SPSS software (https://www.ibm.com/analytics/us/en/technology/spss/) and following the methods of Kujur et al. [54, 55].

### DNA extraction and QTL-seq analysis

Fresh leaves of the CSSL3228, NN1138–2 and 349 F_2_ individuals were collected and ground up in liquid nitrogen. DNA was extracted using the CTAB method [56] with minor modifications and was used for both QTL-seq and molecular marker analysis. For QTL-seq, two DNA pools, higher plant height pool (HPH-pool) and lower plant height pool (LPH-pool), were constructed, respectively, by mixing an equal amount of DNA from 20 higher PH (PH = 58.67–88.33 cm) and 20 lower PH (PH = 35.00–64.67 cm) F_2_ individuals according to the average PH of the F_2:3_ families from the 2015 experiment [57]. Pair-end sequencing libraries (read length 100 bp) with insert sizes of approximately 350 bp were prepared for sequencing with an Illumina HiSeq™ PE150 machine. The short reads from the HPH-pool and the LPH-pool were aligned to the Williams 82 reference genome using BWA software [58]. SNP-calling was performed using the SAM tools software [59]. Low-quality SNPs with a base quality value < 20 were excluded because these SNPs may be false positives due to a genomic repeat sequence or sequencing or alignment errors. Two parameters, SNP-index and Δ (SNP-index) [23, 26], were calculated to identify candidate genomic regions related to PH. The SNP-index is the proportion of reads harboring the SNP that are different from the reference sequence. Δ (SNP-index) was obtained by subtraction of the SNP-index of the HPH-pool from that of the LPH-pool. Thus, SNP-index = 0 if the entire short reads contain genomic fragments from NN1138–2; SNP-index = 1 if all the short reads were from N24852.

An average SNP-index of the SNPs in each genomic interval was calculated using a sliding window analysis with a 1 Mb window size and 1 kb increment. The SNP-index graphs for the HPH-pool and LPH-pool and corresponding Δ (SNP-index) Manhattan graph were plotted. The Δ (SNP-index) value should not be significantly different from 0 in a genomic region with no major QTLs of the target gene. We calculated statistical confidence intervals of Δ (SNP-index) for all SNP positions with given read depths under the null hypothesis of no QTLs and plotted them along with Δ (SNP-index). For each read depth, 95% confidence intervals of Δ (SNP-index) were obtained following [59].

By using Illumina high-throughput sequencing, a total of 207,558,487 and 195,789,024 short reads (150 bp in length) were generated for the HPH-pool (10× depth coverage or 98.44% coverage) and LPH-pool (10× depth coverage or 98.27% coverage), respectively. These short reads were aligned to the Williams 82 reference genome with a size of 978,495,272 bp, and the match rates were between 98.04–98.44% of the reference genome (excluding the N region) among samples covering an average depth of between 9.51× − 29.23 ×, with 1× coverage (at least one covering basis) in more than 94.68%. To identify the candidate genomic region affecting PH, a total of 20,084 polymorphic markers were selected to calculate the SNP-index of HPH and LPH based on the genotyping results. The SNP-index represents the frequencies of parental alleles in the population of bulked individuals. In this case, the NN1138–2 genome was used as the reference, where a SNP-index = 1 indicated that reads in the population were derived only from the NN1138–2 genome and a SNP-index = 0 indicated that the reads were derived only from another parent. A SNP-index of 0.5 indicated an equal genome contribution from both parents. A significant deviation from a SNP-index of 0.5 could indicate the contribution of that SNP to the phenotypic difference observed in the bulks [23].

### SSR marker analysis and QTL verification and fine mapping

To validate and fine map the higher PH QTLs identified by QTL-seq, traditional QTL mapping was carried out. A total of 112 SSR markers in the predicted region on chromosome 13 were selected to survey the polymorphism between the two parental lines according to the integrated soybean genetic linkage map. Among them, 8 polymorphic SSR markers were applied to genotype the F_2_ population. Polymerase chain reaction (PCR) was conducted according to Panaud et al. (1996) with minor modifications. PCR products were separated by 8% PAGE gel to detect polymorphisms [60].

The JoinMap v4.0 software [61] was used to construct the linkage map. To determine the marker order within a linkage group (LG), the JoinMap parameters were set at Rec = 0.40 at different LOD values for different groups. Map distance was converted to centiMorgans (cMs) using the Kosambi mapping function. The linkage map was drawn using Map Chart software [62]. QTL analysis was conducted using the software IciMapping 2.0 using the RSTEP-LRT-ADD model (ICIM mapping) procedure [63–65].

### Expression analysis of candidate genes

To analyze the candidate genes related to PH, we investigated the expression pattern of the genes in the overlap region of QTL-seq and mapping of PH by using real-time quantitative PCR (RT-qPCR). In the summer of 2016, leaf (LE), stem (ST) and shoot apical meristem (SAM) samples at four growth stages—V1, V3, R1 and R2—were collected from P1 and P2. Total RNA for all the samples was extracted using the EasyPure Plant RNA Kit (TranGen Biotech, Beijing, China). Reverse transcription was conducted by Transcript two-step gDNA Removal, and cDNA was synthesized using the Prime Script™ RT Reagent Kit (TaKaRa, Japan) using a standard protocol. The soybean *β-tubulin* gene (Tubulin) was used as an internal control (forward, 5′- GGAGTTCACAGAGGCAGAG’, and reverse, 5′- CACTTACGCATCACATAGC-3′). Tubulin was used as a positive control to ensure the quality of the RNA and cDNA previously. Each step was repeated three times (technical replications). Average relative expression levels for P1 and P2 were calculated. T tests were performed to test the significance of differences in expression levels among different samples. The primers for qRT-PCR were designed by Primer Premier 5.0. To ensure specific amplification, the primer sequences were blasted on the Phytozome v12.1 database (http://www.phytozome.net/soybean). The oligo nucleotides of the RT-qPCR primers are listed in Additional file 10: Table S6.

### Sequence analysis of candidate genes

In order to clarify the mutation of the differentially expressed genes in the two parents in the quantitative analysis, we sequenced the CDS sequence of this five candidate genes(*Glyma.13 g248800*, *Glyma.13 g249000*, *Glyma.13 g249300*, *Glyma.13 g249400* and *Glyma.13 g2496000*). Total RNA was extracted by using the EasyPure Plant RNA Kit (TranGen Biotech, Beijing, China) from leaves of P1(CSSL3228) and P2(NN1138–2). Reverse transcription was conducted by Transcript two-step gDNA Removal, and cDNA was synthesized by using the Prime Script™ RT Reagent Kit (TaKaRa, Japan). The primers for qRT-PCR were designed by Primer Premier 5.0. The target gene was subjected to PCR by using Phanta® Max Super-Fidelity DNA Polymerase from Vazyme, and sent to Kingsley for sequencing (Additional file 11: Table S7) after agarose gel electrophoresis. Amino acid homology alignment using DNAMAN software.

## Additional files

Additional file 1: **Table S1.** Variance analysis for plant height in 2 environments. (XLSX 10 kb)

Additional file 2: **Figure S1.** Distribution of the LOD scores for days to flowering in the target region detected by QTL-seq. (PDF 37 kb)

Additional file 3: **Table S2.** Relative expression level of 12 annotated genes in leaves, stems and shoot apical meristems of NN1138–2 and CSSL3228 at four growth developmental stages. (XLSX 22 kb)

Additional file 4: **Figure S2.** Multiple sequence alignment depicting the amino acid sequence conservation of P1 (CSSL3228) *Glyma.13 g248800* gene with P2 (NN1138–2) gene (*Glyma.13 g248800*) in soybean. (PDF 242 kb)

Additional file 5: **Figure S3.** Multiple sequence alignment depicting the amino acid sequence conservation of P1 (CSSL3228) *Glyma.13 g249300* gene with P2 (NN1138–2) gene (*Glyma.13 g249300*) in soybean. (PDF 303 kb)

Additional file 6: **Figure S4.** Multiple sequence alignment depicting the amino acid sequence conservation of P1 (CSSL3228) *Glyma.13 g249600* gene with P2 (NN1138–2) gene (*Glyma.13 g249600*) in soybean.. (PDF 96 kb)

Additional file 7: **Table S3.** QTL conferring morphological and yield-related traits detected by linkage. (XLSX 12 kb)

Additional file 8: **Table S4.** QTL conferring seed quality traits detected by linkage mapping. LA concentration represents linolenic acid concentration, and ALA represents Alpha-linolenic acid. (XLSX 12 kb)

Additional file 9: **Table S5.** Resistances to diseases and pests, tolerance to stresses and physiological traits. RDT2 represents root distribution in thickness classification 2 (0.5–1.0 mm), and RDL3 represents root distribution in length. (XLSX 11 kb)

Additional file 10: **Table S6.** The oligo nucleotides of the RT-qPCR primers for candidate genes. (XLSX 10 kb)

Additional file 11: **Table S7.** The oligo nucleotides of the cloning primers for candidate genes. (XLSX 10 kb)

## Abbreviations

ANOVA: Analysis of variance
BSA: Bulked-segregant analysis
CSSLs: Chromosome segment substitution lines
HPH-pool: Higher plant height pool
LPH-pool: Lower plant height pool
NGS: Next-generation sequencing
PCR: Polymerase chain reaction
PH: Plant height
QTL: Quantitative trait locus
RT-qPCR: Real-time quantitative PCR
SSR: Simple sequence repeats
