# Supplementary material for: Combining QTL-seq and linkage mapping to fine map a wild soybean allele characteristic of greater plant height
Source: BMC Genomics. 2018 Mar 27;19:226. doi: 10.1186/s12864-018-4582-4 (PMC5870336; doi:10.1186/s12864-018-4582-4)
Supplement: Supplementary file 2 — Figure S1. Distribution of the LOD scores for days to flowering in the target region detected by QTL-seq. (PDF 37 kb) [file 12864_2018_4582_MOESM2_ESM.pdf]

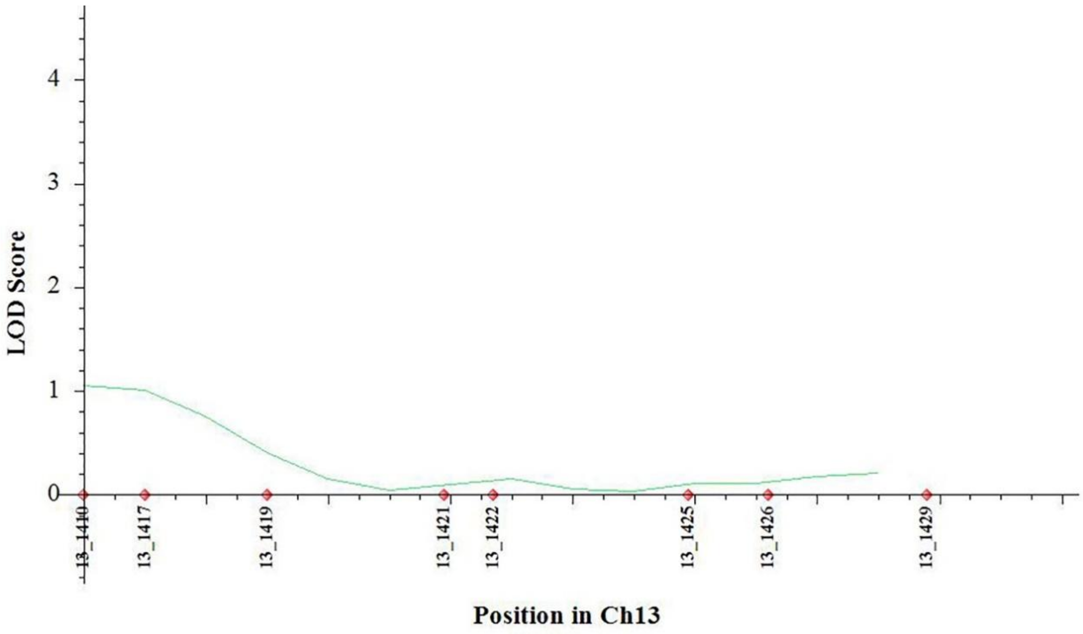

Fig. S1 Distribution of the LOD scores for days to flowering in the target region detected by QTL-seq.
