# Supplementary material for: Combining QTL-seq and linkage mapping to fine map a wild soybean allele characteristic of greater plant height
Source: BMC Genomics. 2018 Mar 27;19:226. doi: 10.1186/s12864-018-4582-4 (PMC5870336; doi:10.1186/s12864-018-4582-4)
Supplement: Supplementary file 4 — Figure S2. Multiple sequence alignment depicting the amino acid sequence conservation of P1 (CSSL3228) Glyma.13 g248800 gene with P2 (NN1138–2) gene (Glyma.13 g248800) in soybean. (PDF 242 kb) [file 12864_2018_4582_MOESM4_ESM.pdf]

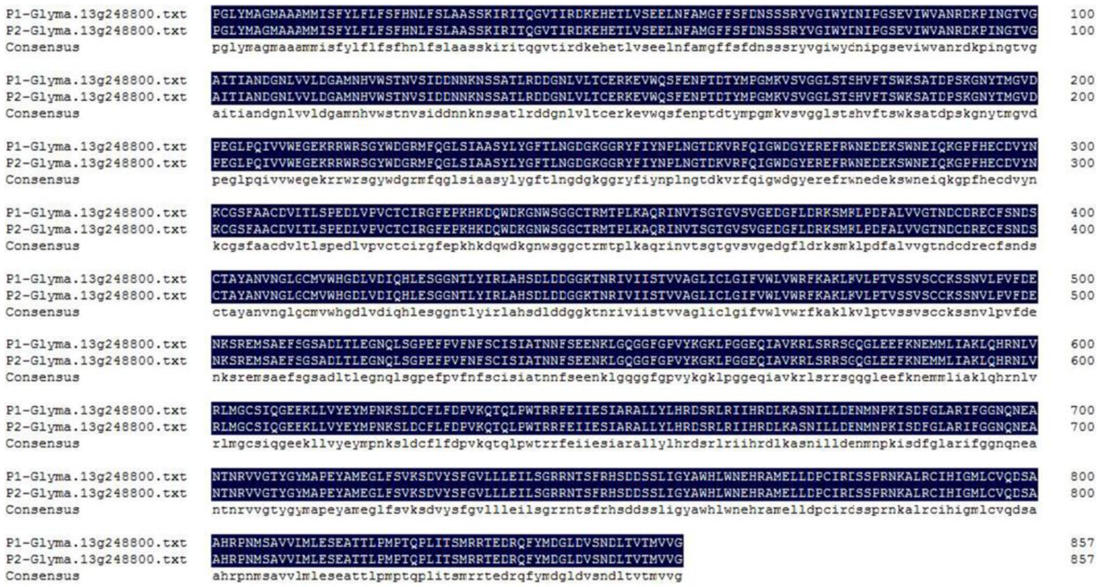

Fig. S2 Multiple sequence alignment depicting the amino acid sequence conservation of P1 (CSSL3228) *Glyma.13g248800* gene with P2 (NN1138-2) gene (*Glyma.13g248800*) in soybean
