# Supplementary material for: Combining QTL-seq and linkage mapping to fine map a wild soybean allele characteristic of greater plant height
Source: BMC Genomics. 2018 Mar 27;19:226. doi: 10.1186/s12864-018-4582-4 (PMC5870336; doi:10.1186/s12864-018-4582-4)
Supplement: Supplementary file 6 — Figure S4. Multiple sequence alignment depicting the amino acid sequence conservation of P1 (CSSL3228) Glyma.13 g249600 gene with P2 (NN1138–2) gene (Glyma.13 g249600) in soybean.. (PDF 96 kb) [file 12864_2018_4582_MOESM6_ESM.pdf]

```
P1-Glyma.13g249600.txt   PGLYMAGMATKVYIVYYSTYGHVEKLAREIEKGAASVEGVEAKLWQVPETLPEEVLAKLGAPPKSDVPIITPNELPEADGFLFGFPTRFGSMAAQFKAFF   100
P2-Glyma.13g249600.txt   PGLYMAGMATKVYIVYYSTYGHVEKLAREIEKGAASVEGVEAKLWQVPETLPEEVLAKLGAPPKSDVPIITPNELPEADGFLFGFPTRFGSMAAQFKAFF   100
Consensus                pglymagmatkvyivyystyghveklareiekgaasvegveaklwqvpetlpeevlaklgappksdvpiitpnelpeadgflfgfptrfgsmaaqfkaff

P1-Glyma.13g249600.txt   DATGGLWRTQALAGKAAGFFYSTSSQGGGQETTPLTSITQLVHHGLIFVPIGYTFGGGMFELEKVKGGSPYGAGTYAGDGSRQPSELELAQAFHQGKYFA   200
P2-Glyma.13g249600.txt   DATGGLWRTQALAGKAAGFFYSTSSQGGGQETTPLTSITQLVHHGLIFVPIGYTFGGGMFELEKVKGGSPYGAGTYAGDGSRQPSELELAQAFHQGKYFA   200
Consensus                datgglwrtqalagkaagffystssqgggqettpltsitqlvhhglifvpigytfgggmfelekvkggspygagtyagdgsrqpselelaqafhqgkyfa

P1-Glyma.13g249600.txt   GIAKKLKGS                                                                                              209
P2-Glyma.13g249600.txt   GIAKKLKGS                                                                                              209
Consensus                giakklkgs
```

Fig. S4 Multiple sequence alignment depicting the amino acid sequence conservation of P1 (CSSL3228) *Glyma.13g249600* gene with P2 (NN1138-2) gene (*Glyma.13g249600*) in soybean.
